# Supplementary material for: Identification of photoperiod-induced specific miRNAs in the adrenal glands of Sunite sheep (Ovis aries)
Source: Front Vet Sci. 2022 Jul 22;9:888207. doi: 10.3389/fvets.2022.888207 (PMC9354845; doi:10.3389/fvets.2022.888207)
Supplement: Supplementary file 1 [file Data_Sheet_1.ZIP › Supplementary file/miRDeep2 file.zip!/miRDeep2 file/Know_Structure/LP42A3/oar-mir-543.pdf]

miRBase precursor : oar-mir-543
Total read count : 19603
oar-miR-543-5p read count : 8
oar-miR-543-3p read count : 19524
remaining reads : 71

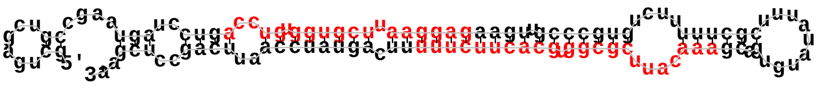

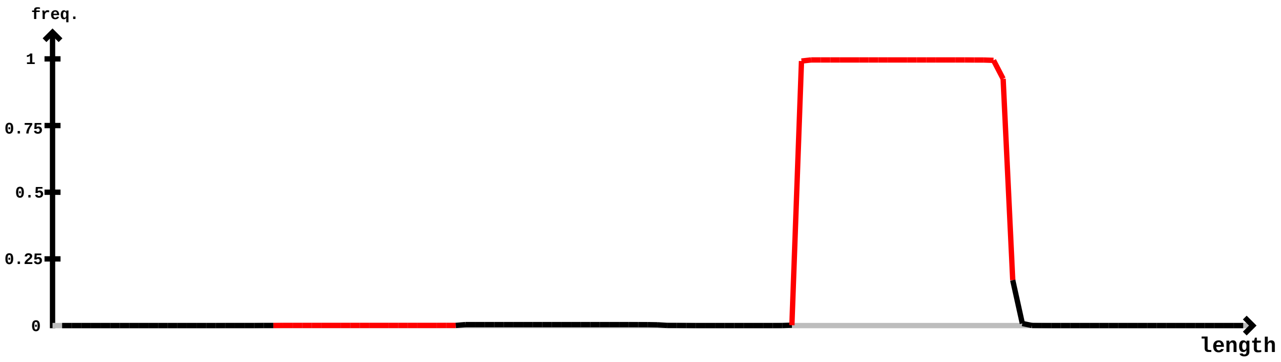


```
                                                                                         oar-miR-543-3p
                        oar-miR-543-5p
5'- gcugagcugccgaaugauccugaccuguggugcuuaaggagaaguugcccgugucuuuuucgcuuuauaugugacgaaacauucgcggugcacuucuuuuucaguauccaauucagccucgaa  -3'   exp
    ((......)).....((..((((..((.((((((.((((((((((.(((((((....((((((.........).)))))....))))).)))))))))))).))))))))..))))..))...   reads      mm
    ................auccugaccuguggugcuuaagg....................................................................................   1          0
    ....................ugaccuguggugcuuaagg....................................................................................   1          0
    ....................ugaccuguggugcuuaagga...................................................................................   4          0
    ......................accuguggugcuuaagga...................................................................................   2          0
    ......................accuguggugcuuaaggag..................................................................................   1          0
    .......................................agaaguugcccgugucuuuu................................................................   1          0
    .......................................agaaguugcccgugucuuuuuc..............................................................   1          0
    .......................................agaaguugcccgugucuuuuucg.............................................................   2          0
    ........................................gaaguugcccgugucuuuuuc..............................................................   1          0
    ........................................gaaguugcccgugucuuuuucg.............................................................   3          0
    .........................................aaguugcccgugucuuuuu...............................................................   3          0
    .........................................aaguugcccgugucuuuuuc..............................................................   9          0
    .........................................aaguugcccgugucuuuuucg.............................................................   41         0
    .........................................aaguugcccgugucuuuuucgc............................................................   1          0
    .........................................aaguugcccgugucuuuuucgcu...........................................................   5          0
    .........................................aaguugcccgugucuuuuucgcuu..........................................................   3          0
    ..........................................................................cgaaacauucgcggugcacuucu..........................   2          0
    ...........................................................................gaaacauucgcggugcacuucu..........................   24         0
    ...........................................................................gaaacauucgcggugcacuucuu.........................   2          0
    ............................................................................aaacauucgcggugcacu.............................   3          0
    ............................................................................aaacauucgcggugcacuu............................   3          0
    ............................................................................aaacauucgcggugcacuuc...........................   22         0
    ............................................................................aaacauucgcggugcacuucu..........................   1332       0
    ............................................................................aaacauucgcggugcacuucuu.........................   14751      0
    ............................................................................aaacauucgcggugcacuucuuu........................   3156       0
    ............................................................................aaacauucgcggugcacuucuuuu.......................   144        0
    ............................................................................aaacauucgcggugcacuucuuuuu......................   4          0
    ............................................................................aaacauucgcggugcacuucuuuuuc.....................   1          0
    .............................................................................aacauucgcggugcacuucuu.........................   48         0
    .............................................................................aacauucgcggugcacuucuuu........................   29         0
    .............................................................................aacauucgcggugcacuucuuuu.......................   3          0
```
